# Diagnostic utility of *BRAF^V600E ^*mutation testing in thyroid nodules in elderly patients

**DOI:** 10.1186/1471-2482-13-S2-S37

**Published:** 2013-10-08

**Authors:** Anna Guerra, Vincenzo Di Crescenzo, Alfredo Garzi, Mariapia Cinelli, Chiara Carlomagno, Stefano Pepe, Pio Zeppa, Massimo Tonacchera, Mario Vitale

**Affiliations:** 1 Department of Medicine and Surgery, University of Salerno, Salerno, Italy; 2 Department of Public Health, University of Naples "Federico II", Naples, Italy; 3 Department of Clinical Medicine and Surgery, University of Naples "Federico II", Naples, Italy; 4 Department of Endocrinology, Research Center of Excellence AmbiSEN, University of Pisa, Pisa, Italy

## Abstract

**Background:**

Thyroid cancer is a rare disease characterized by the subtle appearance of a nodule. Fine-needle cytology (FNC) is the first diagnostic procedure used to distinguish a benign from a malignant nodule. However, FNC yields inconclusive results in about 20% of cases. *BRAF^V600E ^*mutation is the most frequent genetic alteration in papillary thyroid carcinoma (PTC); its high prevalence makes this oncogene a useful marker to refine inconclusive FNC results. However, the prevalence of the *BRAF^V600E ^*mutation depends on detection methods, geographical factors, and age. The aim of this study is to determine the prevalence of *BRAF^V600E ^*mutation and its utility as a diagnostic tool in elderly subjects.

**Methods:**

FNC from 92 PTC patients were subjected to the analysis of *BRAF *mutation by pyrosequencing and direct sequencing; age-dependent prevalence was also determined.

**Results:**

*BRAF *mutation analysis was successful in all FNC specimens. *BRAF^V600E ^*was documented in 62 (67.4%) and in 58 (63.0%) PTCs by pyrosequencing and direct sequencing, respectively. *BRAF^V600E ^*prevalence did not correlate with patient's age at diagnosis. Twenty out of 32 PTCs (62.5%) were correctly diagnosed by *BRAF *mutation analysis in inconclusive FNC results.

**Conclusions:**

Detection of *BRAF^V600E ^*in cytology specimens by pyrosequencing is a useful diagnostic adjunctive tool in the evaluation of thyroid nodules also in elderly subjects.

## Introduction

Living in an oxygenated environment has required the evolution of effective cellular strategies to detect and detoxify metabolites of molecular oxygen known as reactive oxygen species. Reactive oxygen species (ROS) are highly reactive molecules that consist of a number of diverse chemical species including superoxide anion (O2*-*), hydroxyl radical (*·*OH), and hydrogen peroxide (H2O2) [1]. Oxidative stress is an important aspect of cancer, diabetes, neurodegenerative, cardiovascular and other diseases, and elevated ROS has been implicated in the mechanism of senescence and aging [2,3]. Oxidant overproduction occurs in response to several stressors, including chemicals, drugs, pollutants, high-caloric diets and exercise [4]. The prevalence of palpable thyroid nodules in iodine-sufficient regions ranges between 1% - 9% in adults [5]. It is lower in young people and increases progressively with age. However, the prevalence of nodular goiter markedly increases when ultrasonography is used. About 75% of individuals over the age of 80 years have nodules on ultrasound examination. The majority of thyroid nodules are benign and the incidence of thyroid cancer is low, accounting for about 5% of nodules [6,7], although it has increased over the last decades. The increase of incidence is not equally attributed to all types of thyroid cancer. Papillary thyroid carcinoma (PTC) is the most frequent thyroid cancer, accounting for approximately 85 - 90% of all thyroid cancers, whereas follicular thyroid carcinoma accounts for about 10% or less, and poorly differentiated and undifferentiated or anaplastic carcinomas are very rare (approximately 1 - 2%) [8]. The risk of thyroid cancer is higher in women and people with low iodine intake, high body mass index and radiation exposure [9-11]. Hashimoto's Thyroiditis (HT) is a frequent thyroid disorder whose prevalence increases with age. PTC is believed to be more frequent in patients with concurrent HT. *RET *rearrangements (*RET/PTC*) and *BRAF *point mutations are genetic alterations occurring in PTC, proposed as tumor markers to refine inconclusive FNC results. The identification of changes in the expression of other molecular biomarkers, such as Ca^2+^-transporting proteins, which have been proposed as an alternative means for tumor diagnosis [12-19], is still missing. *BRAF^V600E ^*is the most frequent mutation in PTC and, unlike *RET/PTC*, it has never been detected in benign thyroid nodules. Its utility as a diagnostic marker depends upon its prevalence, which varies on the basis of detection methods and geographical factors. While *RET *rearrangements are induced by thyroid exposure to ionizing radiations, no *BRAF *mutation inducing factors have been identified so far. The higher prevalence of age-related thyroid diseases, such as HT or longer exposure to endocrine disrupters and thyroid toxic agents, may affect the prevalence of *BRAF *mutations in elderly subjects as well as the sensitivity of *BRAF^V600E ^*as a tumor marker. The clinical appearance of thyroid cancer is that of a nodules, some time representing a challenging diagnostic dilemma with thyroid or unusual extrathyroidal masses [20,21]. Until serological biomarkers are available, FNC is the primary diagnostic tool offering the highest values of sensitivity and specificity [22-27]. Nonetheless, inconclusive results may occur and the application of molecular techniques to FNC has dramatically increased its sensitivity [25,28-31], including in the case of HT with diffuse or nodular enlargement [26]. These advantages are enhanced in the case of benign nodules, which does not require surgical treatment, and even more in the elderly, where surgery is generally more burdensome, complex and expensive than in younger patients [32,33]. Hence, the aims of the present study are to determine the utility of *BRAF^V600E ^*mutation detection as a diagnostic tool to refine inconclusive FNC results in elderly subjects and whether its sensitivity is age-related.

### Material and methods

#### Patients and clinicopathological data collection

A total of 92 patients with PTC were enrolled in the study after giving their consent and with the approval from the institutional review board. Clinicopathological data included: age at diagnosis, gender, tumor size, and TNM staging. Tumor volume was calculated according to the formula of the ellipsoid model: volume (mL) = width × length × thickness × □/6. After surgical resection, tissues were fixed in formalin, embedded in paraffin wax, and stained with haematoxylin and eosin for microscopy studies. Standard criteria were employed to classify tumors and their variants [34]. FNC was performed and classified according to the British Thyroid Association [35] as described elsewhere [36,37]. As far as the concomitant lymphoid infiltrate concerns, its polyclonal, inflammatory nature was assessed in selected cases by flow cytometry (FC) and related data were interpreted accordingly [38-41] in this specific clinical and anatomical setting. Clinicopathological data are reported in Table 1.

**Table 1:** Pre-surgical clinicopathological features of PTC patients with concurrent HT.

	Age (years)		P
**Clinical features**	**< 70**	**≥ 70**	

Number of patients	74	18	
Age at diagnosis years, mean, range	79.4, 70-88	50.4, 21-67	
Gender (male)	29.8%	33.3%	n.s.
Substitutive therapy with L-T4	41.8%	33.3%	n.s.
Tumor volume mL, mean, range	1.4, 1.0-22.2	2.5, 1.5-5.5	n.s.
Multinodularity	75%	78%	n.s.
Cervical lymphadenopathy^a^	7	2	n.s.
Neck irradiation	0	1	
Symptoms of compression	1	0	
Fast growth^b^	0	0	
Symptoms of infiltration	0	0	
Familiarity for thyroid carcinoma	1	0	

^a^, detected by ultrasonography; ^b^, doubling of the tumor volume in 1 year; n.s., not significant

#### DNA extraction from cytology samples

Cytology samples were obtained using a syringe with a 22-gauge needle passed three to four times. Material from the needle was used to prepare a smear for cytology, then the needle was washed out with 5 ml of normal saline into a collection tube, and centrifuged. The pellet was resuspended into TRI Reagent buffer (Sigma) and stored at -20 °C for DNA extraction.

#### Detection of the *BRAF *mutation

Direct sequencing was performed by BigDye Terminator method. DNA was amplified by polymerase chain reaction (PCR) with specific primers, as described previously [42]. Pyrosequencing was performed as described in detail elsewhere [42,43]. Briefly, DNA was amplified by PCR, processed to obtain single-stranded DNA, hybridized to sequencing primers, and sequencing-by-synthesis reaction of the complementary strand was automatically performed on a PSQ 96MA instrument (Biotage, Uppsala, Sweden). The cut-off was set at 5%, corresponding to the mean percentage of normal tissues plus 2 SD.

#### Statistics

Results were analyzed by the chi-square of independence test or the t-test with Prism (Version 3.00 for Windows; GraphPad Software, San Diego, CA, USA). The level of significance was set at *P *< 0.05.

### Results

A total of 92 PTC patients entered the study. Patients were divided into 2 groups: < 70 years old (No. = 74) and ≥ 70 years old (No. = 18). The mean age of the 2 groups was 79.4 and 50.4, respectively. Cervical lymphadenopathy was documented by ultrasonography in 9 subjects; neck irradiation, symptoms of compression and familiarity for thyroid carcinoma were documented in 1 patient, respectively (Table 1). *BRAF *mutational analysis was successful in all cytology specimens. *BRAF^V600E ^*was documented in 62 patients (67.4%) and in 58 (63.0%) patients by pyrosequencing and direct sequencing, respectively (Table 2). The prevalence of *BRAF^V600E ^*mutation in the 2 age groups was similar (67.6 *vs*. 66.6 by pyrosequencing in < 70 years *vs*. ≥70 years, respectively). The mean age of PTC patients (*BRAF^V600E ^*positive or negative) was similar (54.4 and 54.3 respectively, P= 0.461). Twenty-four (26.1%) FNCs yielded inconclusive results (Table 3), 10.9% and 13.4% were THY3 and THY4, respectively. *BRAF^V600E ^*mutation was documented in 16 inconclusive FNC results (66.6%). No significant difference in sensitivity was documented between young and elderly patients.

**Table 2:** Prevalence of *BRAF^V600E ^*mutation in 92 PTCs.

Age (years)	*Direct Sequencing*	*Pyrosequencing*
<70	11 (61.1)	12 (66.6)
≥70	47 (63.5)	50 (67.6)
P	n.s.	n.s.

Number of positive samples (and percentages).

n.s., not significant

**Table 3:** FNC results and detection of *BRAF^V600E ^*mutation by pyrosequencing.

FNC	Age, > 70 years		Age, ≥ 70 years	
**TYH**	**total **	***BRAF^V600E ^***	**total**	***BRAF^V600E ^***

3	8	6 (75.0)	2	1 (50.0)
4	11	7 (63.6)	3	2 (66.6)
5	55	37 (67.2)	13	9 (69.2)

Number of positive samples (and percentages).

TYH 3, indeterminate; TYH4, suspicious for malignancy; TYH5, malignant

### Discussion

The clinical utility of molecular testing for inconclusive FNC results was demonstrated by a number of studies in the last decade. Among the genetic alterations known in thyroid cancer, *BRAF^V600E ^*is the most useful to this purpose, because of its high sensitivity and its absolute specificity. However, its sensitivity depends on the detection method and on its prevalence. cDNA sequencing is the gold standard for the detection of genetic mutations. However, the sensitivity of these methods is reduced by the diluting effect of the wild type gene carried by tumor cells and non-tumor cells in the specimen [23]. This is particularly relevant when other thyroid diseases are present. Thyroid autoimmunity, and HT in particular, are frequent in the general female population and even more frequent in the elderly. These diseases are characterized by an abundant lymphoplasmacytic infiltrate of the thyroid and in the late stages by fibrous atrophy. Hence, the sensitivity of molecular tests applied to thyroid cytology specimens was hypothesized to be reduced in elderly subjects [44]. Another possible factor affecting the sensitivity of *BRAF^V600E ^*diagnostic tests is the prevalence of this oncogene in a specific cohort. Besides the detection methods, racial or geographical factors are the major determining factors of *BRAF^V600E ^*prevalence. This oncogene is much more frequent in the Korean population than in any other, being detected in about 90% of PTCs. Its high prevalence can be the result of a particular genetic imprinting or the effect of food or environmental factors. Iodine diet content and goitrogen factors, the exposure to ionizing radiations, endocrine disrupters and thyroid toxic agents can be relevant in the development of thyroid cancer. A low iodine diet has been associated with a higher prevalence of thyroid cancer, with a higher papillary/follicular thyroid ratio [45]. Also, the exposure to ionizing radiations is a risk factor for the development of thyroid cancer. The genetic rearrangement of the proto-oncogene *RET *generates chimeric proteins (RET/PTC) with demonstrated carcinogenetic properties. RET rearrangements, and *RET/PTC3 *in particular, are induced by the exposure to ionizing radiations, so that their prevalence is increased in the exposed populations [46,47]. Time of exposition to interfering agents is crucial for carcinogenesis, so that the risk of thyroid cancer development is age-dependent. Studies on population of different geographic areas showed the reduction of *RET/PTC *in thyroid tumors in older subjects [48,49]. Although *BRAF^V600E ^*has been extensively investigated, evidence on the etiology of this oncogene is lacking. Study results indicate that the prevalence of *BRAF^V600E ^*does not change in elderly subjects and that it can be used as a PTC marker in inconclusive FNC results. The sensitivity of the Big Dye terminator method is low since it is based on an automated or subjective evaluation of a chromatogram. For this reason, mutations detected in less than 20% of PCR products yield ambiguous or false negative results [50]. Accordingly, *BRAF^V600E ^*detection in this study was higher by pyrosequencing analysis than by direct sequencing, and PTC was correctly diagnosed in 66.6% of FNC inconclusive results by means of this method.

## Conclusions

The prevalence of *BRAF^V600E ^*mutation in PTCs of elderly subjects is similar to that of younger subjects. The detection of *BRAF^V600E ^*mutation in cytology specimens by pyrosequencing is a useful diagnostic adjunctive tool in the evaluation of thyroid nodules also in elderly subjects.

## Competing interests

The authors declare that they have no competing interests.

## Authors' contributions

MV: conception and design, interpretation of data. AG, PZ, VDS, AG, MCP, CC, SP, MT: acquisition of data, drafting the manuscript. PZ, MV: critical revision, given final approval of the version to be published.

## Authors' information

AG = Resident in Clinical Pathology at University of Salerno. VDC = Aggregate Professor of Thoracic Surgery at University of Salerno. AG = Aggregate Professor of Pediatric Surgery at University of Salerno. MC = Aggregate Professor of Anatomy, University of Naples "Federico II". CC = Aggregate Professor of Oncology, University of Naples "Federico II". SP = Associate Professor of Oncology, University of Salerno. PZ = Associate Professor of Pathology at University of Salerno. MT = Associate Professor of Endocrinology at University of Pisa. MV = Associate Professor of Endocrinology at University of Salerno.

## Declarations

Publication charges for this article were covered by research funds of the project Bando Faro 2011 - Finanziamenti per l'Avvio di Ricerche Originali, cofounded by the Compagnia di San Paolo and by the Polo per le Scienze e le Tecnologie per la Vita of the University Federico II in Naples.

This article has been published as part of *BMC Surgery *Volume 13 Supplement 2, 2013: Proceedings from the 26th National Congress of the Italian Society of Geriatric Surgery. The full contents of the supplement are available online at http://www.biomedcentral.com/bmcsurg/supplements/13/S2
